# Supplementary material for: Filamentous Temperature-Sensitive Z Protein J175 Regulates Maize Chloroplasts’ and Amyloplasts’ Division and Development
Source: Plants (Basel). 2025 Jul 16;14(14):2198. doi: 10.3390/plants14142198 (PMC12298180; doi:10.3390/plants14142198)
Supplement: Supplementary file 1 [file plants-14-02198-s001.zip › plants-3719031-supplementary.pdf]

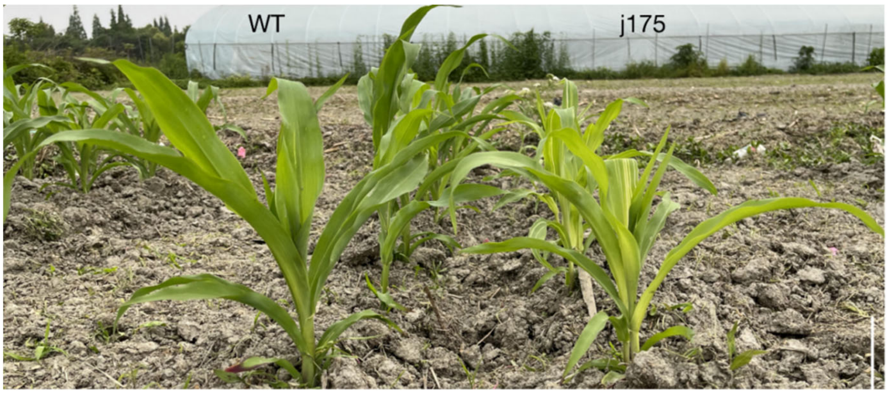

Figure S1 . Comparison of seedling plants. bar, 5cm.

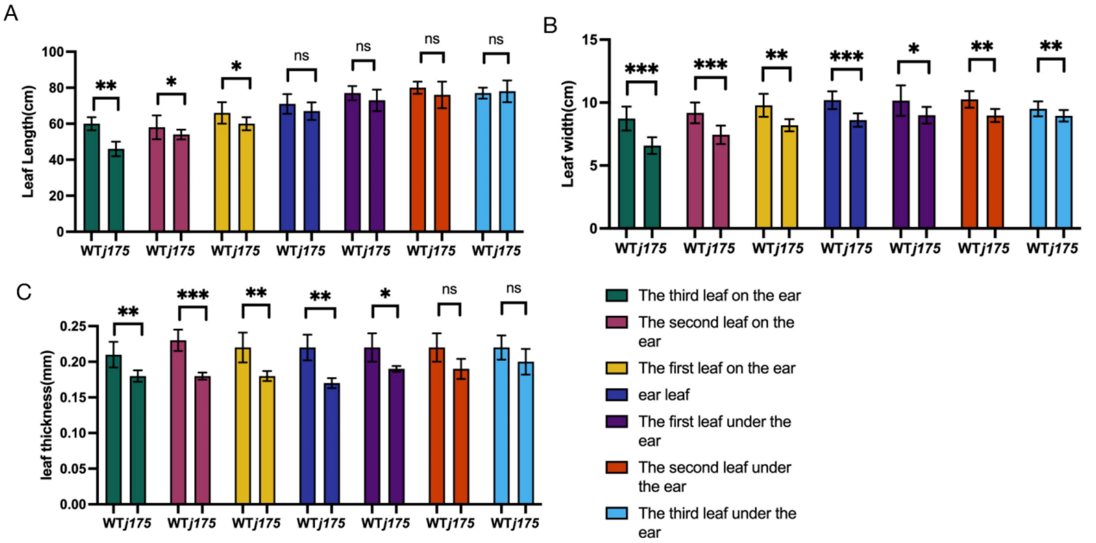

Figure S2 . Comparison of leaf types between *j175* and WT. A, laef length. B, leaf width. C, leaf, thickness.

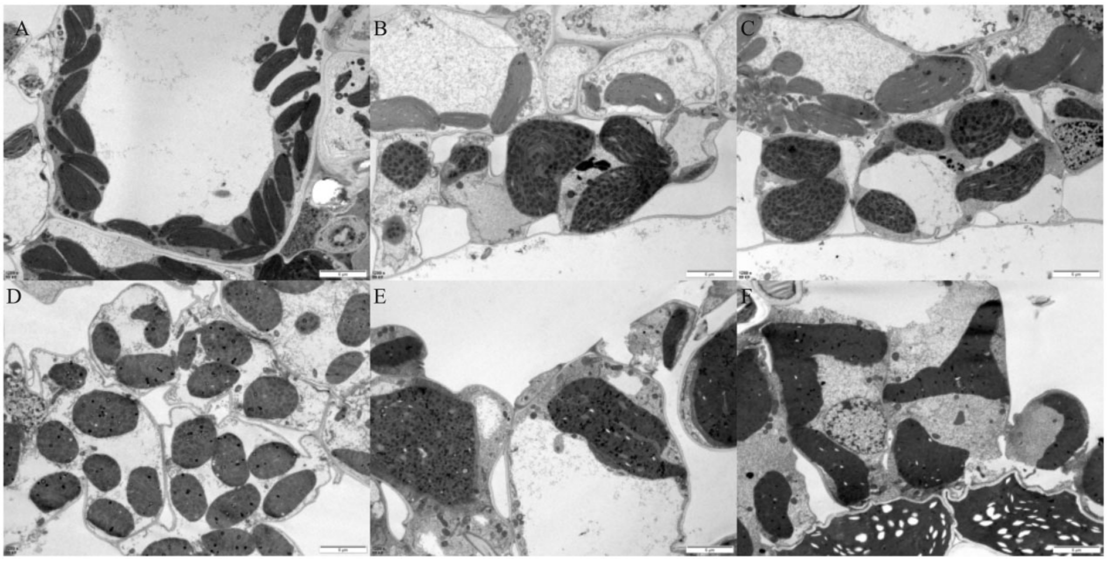

Figure S3. Observation of chloroplasts in mesophyll cells by projection electron microscopy. A, WT leaf mesophyll cells during seedling stage. B-C, *j175* leaf mesophyll cells during seedling stage.D, Mature WT mesophyll cells. E-F,Mature

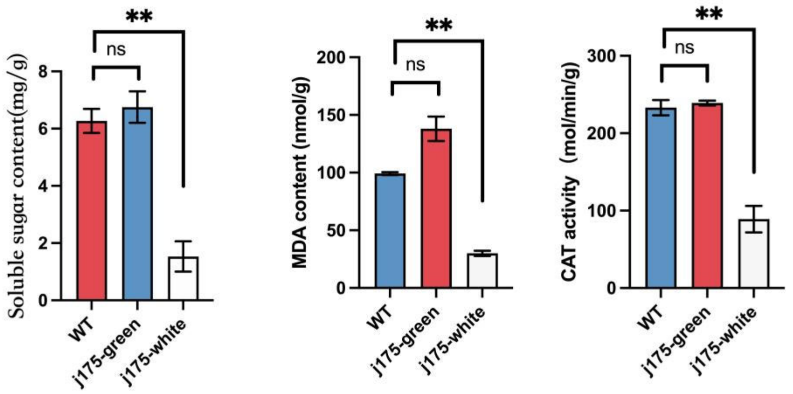

Figure S4. Enzyme activity in j175

Enzyme activity assay: MDA (malondialdehyde), CAT (catalase). Data are presented as mean ± SD and statistically analyzed using Student's t-test, with N=4. * ($p<0.05$), ** ($p<0.01$)

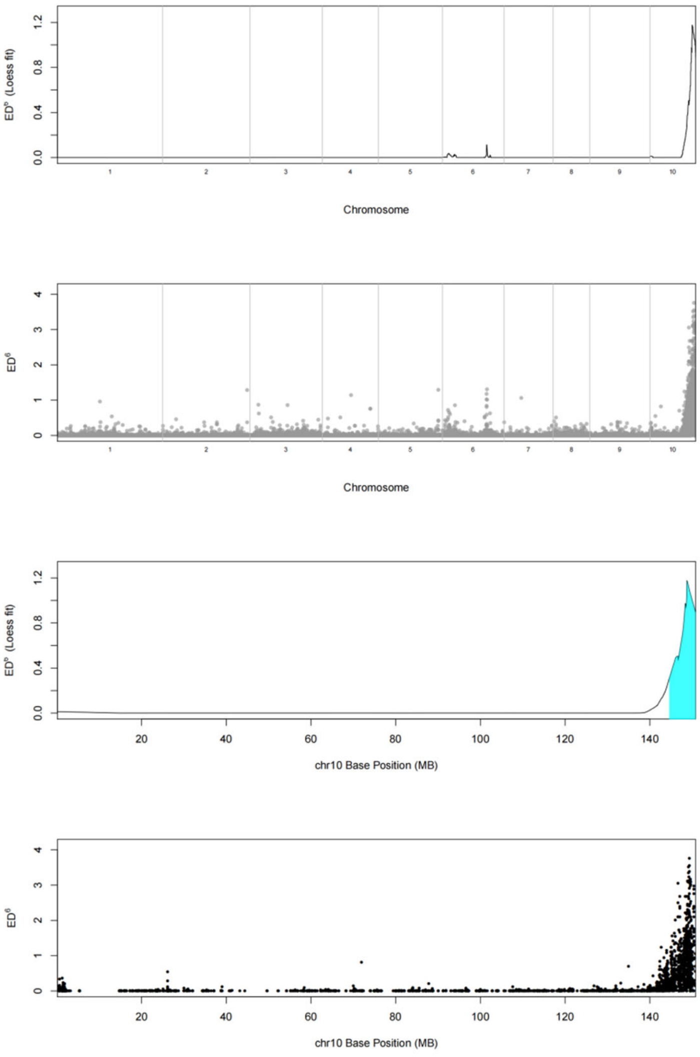

Figure S5. BSA-seq analysis

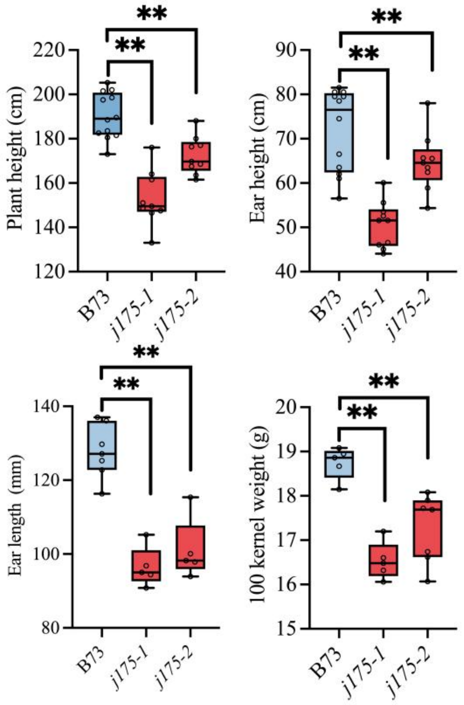

Figure S6. Partial agronomic traits of *j175-1* and *j175-2*.

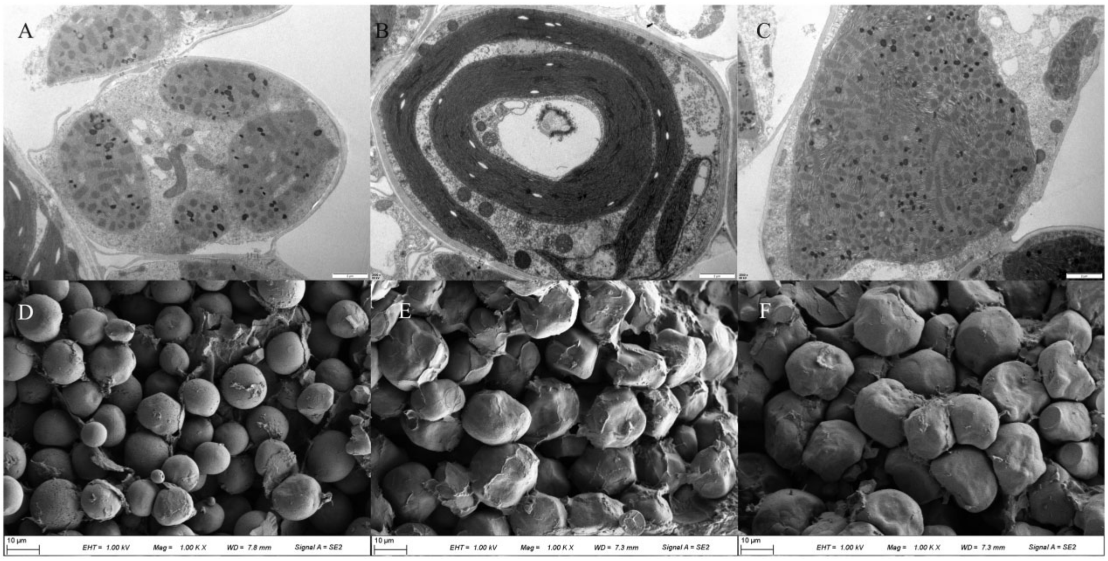

Figure S7. Observation of chloroplast morphology in mesophyll cells of j175-1 and j175-2 by projection electron microscopy and starch granule morphology in mature endosperm cells by scanning electron microscopy

(A) WT mesophyll cell chloroplasts; (B) J175-1 leaf mesophyll cell chloroplasts; (C) J175-2 leaf mesophyll cell chloroplasts; (D) WT endosperm cell starch granules; (E) J175-1 endosperm cell starch granules; (F) J175-2 endosperm cell starch granules.

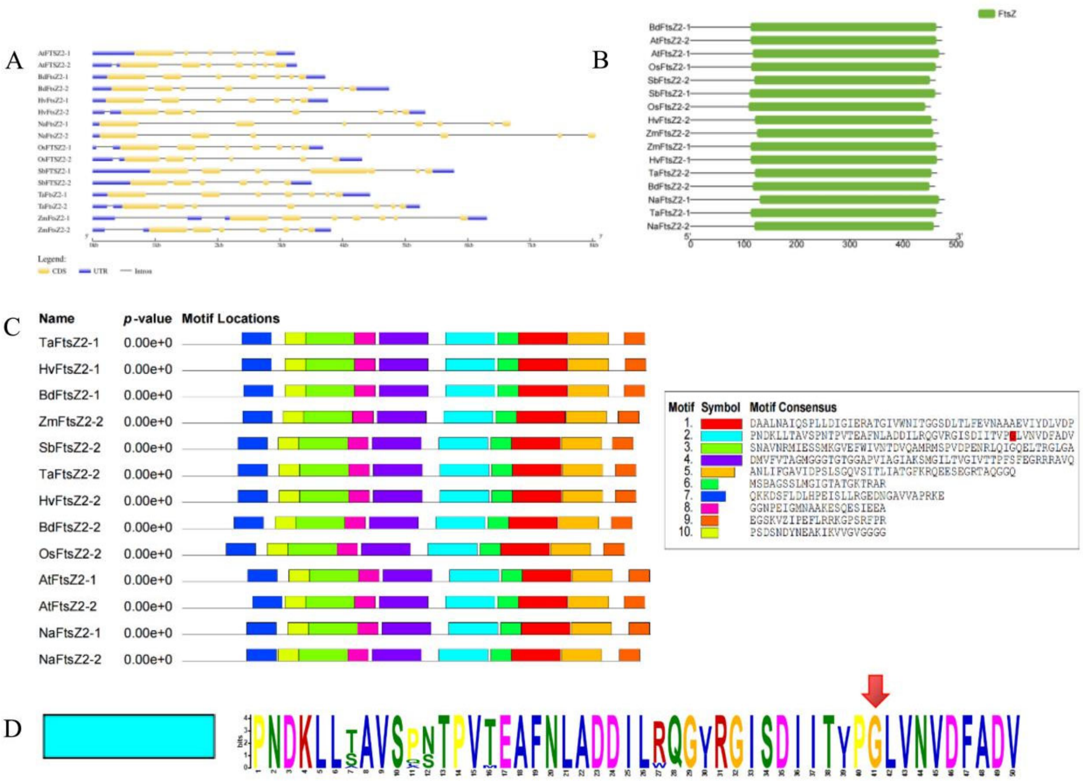

Figure S8. Gene Evolution Analysis. A, Gene structure analysis. B, Conservative structural domain analysis. C–D, Gene motif analysis.

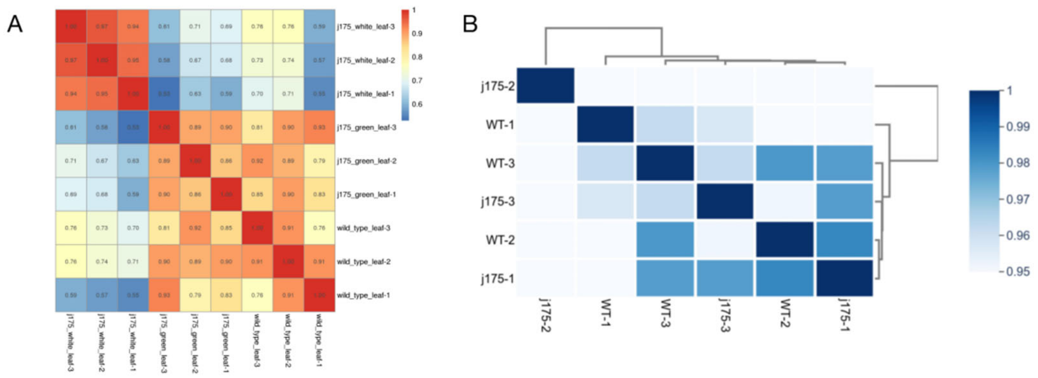

Figure S9. The clustering heatmap analysis of differential genes. A, leaf. B, kernel.

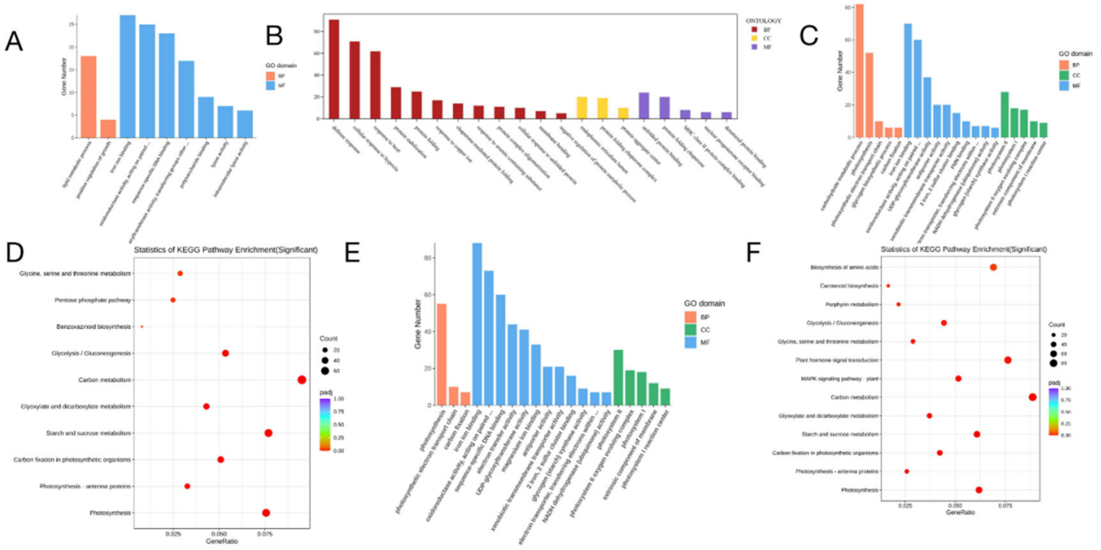

Figure S10. Gene Ontology Categories and Pathway Enrichment of Differentially Expressed Genes.A,GO analysis of WT-Leaf and *j175*-green-leaf.B, GO analysis of *j175*-green-leaf and *j175*-white-leaf. C-D, GO and KEGG enrichment analysis of WT-Leaf and *j175*-white-leaf; E-F, GO and KEGG enrichment analysis of *j175*-green-leaf and *j175*-white-leaf;

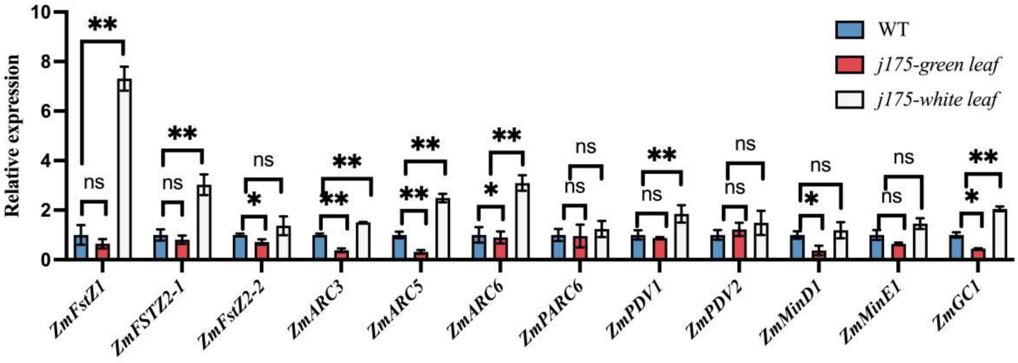

Figure S11. qPCR of plastid division related genes

Table S1. the chi-square test of the F2 population

population	Normal plant	White striped plant	Theoretical proportion	X^2	$X^2_{0.005}$
j175×B73	393	107	3:01	3.27	<3.841

Table S2. Molecular markers used for final-mapped of j175.

molecular markers	F primer	R primer

unc1084	GATAAAAAGGCAAGTGCAACAAGG	ATATCAACCAGAGGCTGGAACTTG
indel1	CTGGTAACACCATCGACGAG	ACGACCTAAACAATAGCCAAC
indel2	TGTAGCGTTGGTGTCAATACT	TCACTGGAAAGGCAAGCTAC
indel3	ACAGAGTAGATAGACCGGCG	ATAGCTAGCTCTCCTGCCG
indel4	TCCGTCACTTGACTAGAGGT	ATAACACAGCTGCCTCACAG
indel5	TGGGCCTATTAGCTCAACATTT	GGTAAGTCCAAGTCCACCAA
indel6	GTCACAGCACATCCGTTACT	CGCGTGGCCAGAAATTTTA
indel7	AGCGGGTTTTAACCCAAGAC	CCAGTGAAGTTCCAACTCTGT

Table S3**. Protein sequence information**

Zea may	*ZmFtsZ2-1*	*Zm00001eb348280*
	ZmFtsZ2-2	*Zm00001eb434230*
Arabidopsis thaliana	*AtFTSZ2-1*	*AT2G36250*
	AtFTSZ2-2	*AT3G52750*
Oryza sativa	*OsFTSZ2-1*	*Os05g0443800*
	OsFTSZ2-2	*Os03g0646032*
Sorghum bicolor	*SbFTSZ2-1*	*SORBI_3009G154900*
	SbFTSZ2-2	*SORBI_3006G275000*
Nicotiana attenuata	*NaFtsZ2-1*	*OIT21422*
	NaFtsZ2-2	*OIT01485*
Triticum aestivum	*TaFtsZ2-1*	*TraesCS1D02G276000*
	TaFtsZ2-2	*TraesCS2D02G292200*
Solanum lycopersicum	*SlFtsZ2-1*	*Solyc10g083490.2*
	SlFtsZ2-2	*Solyc09g009430.3*
Hordeum vulgare	*HvFtsZ2-1*	*HORVU.MOREX.r3.1HG0074730*
	HvFtsZ2-2	*HORVU.MOREX.r3.2HG0166260*
Brachypodium distachyon	*BdFtsZ2-1*	*BRADI_2g23900v3*
	BdFtsZ2-2	*BRADI_5g09300v3*

Table S4. Vector construction and qPCR primers

**Primer name**	**Sequence (5'–3')**
CDSj175-F	GGCATTCGTGGCATATCTGA
CDSj175-R	GCGCAGGGTAAATAAAACGG
CDSj175-p2300KpnI -F	atttggagaggacagggtaccATGGCTACACAGTTGCCATGC
CDSj175-p2300XbaI-R	ggtactagtgtcgactctagaAACTCGTGGGAAGCGAGAAGG
Zmj175-qPCR-F	GTCAAGGCATTCGTGGCATA
Zmj175-qPCR-R	AGCATCCCTTGCTCTTGACT
ZmFtsZ1-qPCR-F	CAAAGGGAGCACGGATAGT
ZmFtsZ1-qPCR-R	TCTTCGAGACCAAGCAGAAG
ZmFtsZ2-1-qPCR-F	GCAAGGTGTTCGTGGGATA
ZmFtsZ2-1-qPCR-R	GAGCCAGCATCTGACATAACT

ZmARC5-qPCR-F	CGTTGTCAAATCTGCTGGTAATC
ZmARC5-qPCR-R	CTGCCTCTACTTGCCTTCTTT
ZmARC5-qPCR-F	ATGTCACTTGGTCCCTTCAC
ZmARC5-qPCR-R	CGAGTCACCCTTTGCCTTAT
ZmARC6-qPCR-F	CCGGTCGACTTCTACAAGATTC
ZmARC6-qPCR-R	CTTCTGTGCTGTACCCATACTG
ZmPARC6-qPCR-F	CGAGAAGGCTGAGAAAGATGAA
ZmPARC6-qPCR-R	CTACTAGGAGAGCCTGTCTGAA
ZmPDV1-qPCR-F	CCTTGCTATGAGGTTAGCAGAA
ZmPDV1-qPCR-R	CGAACATCCTCAGGTGAGATAAA
ZmMinD1-qPCR-F	ACCCGCATTCATCCTCATC
ZmMinD1-qPCR-R	CGTAATGTCGGGAGTGGTAA
ZmMinE1-qPCR-F	TCAAGGGATAACGTCCAAGTG
ZmMinE1-qPCR-R	GCTCTTCAGATTCCTGGTACTC
ZmGC1-qPCR-F	CTTCTGTGGTGTTGGAAGGA
ZmGC1-qPCR-R	ATGGCCTTTAGAGCGTCTTC
